# Cabbage and Weed Identification Based on Machine Learning and Target Spraying System Design

**DOI:** 10.3389/fpls.2022.924973

**Published:** 2022-08-04

**Authors:** Xueguan Zhao, Xiu Wang, Cuiling Li, Hao Fu, Shuo Yang, Changyuan Zhai

**Affiliations:** ^1^Intelligent Equipment Research Center, Beijing Academy of Agriculture and Forestry Sciences, Beijing, China; ^2^National Engineering Research Center of Intelligent Equipment for Agriculture, Beijing, China; ^3^College of Mechanical Engineering, Guangxi University, Beijing, China

**Keywords:** target spraying, independent nozzle control, target identification, effective spraying rate, pesticide saving amount

## Abstract

The complexity of natural elements seriously affects the accuracy and stability of field target identification, and the speed of an identification algorithm essentially limits the practical application of field pesticide spraying. In this study, a cabbage identification and pesticide spraying control system based on an artificial light source was developed. With the image skeleton point-to-line ratio and ring structure features of support vector machine classification and identification, a contrast test of different feature combinations of a support vector machine was carried out, and the optimal feature combination of the support vector machine and its parameters were determined. In addition, a targeted pesticide spraying control system based on an active light source and a targeted spraying delay model were designed, and a communication protocol for the targeted spraying control system based on electronic control unit was developed to realize the controlled pesticide spraying of targets. According to the results of the support vector machine classification test, the feature vector comprised of the point-to-line ratio, maximum inscribed circle radius, and fitted curve coefficient had the highest identification accuracy of 95.7%, with a processing time of 33 ms for a single-frame image. Additionally, according to the results of a practical field application test, the average identification accuracies of cabbage were 95.0%, average identification accuracies of weed were 93.5%, and the results of target spraying at three operating speeds of 0.52 m/s, 0.69 m/s and 0.93 m/s show that the average invalid spraying rate, average missed spraying rate, and average effective spraying rate were 2.4, 4.7, and 92.9%, respectively. Moreover, it was also found from the results that with increasing speeds, the offset of the centre of the mass of the target increased and reached a maximum value of 28.6 mm when the speed was 0.93 m/s. The void rate and pesticide saving rate were 65 and 33.8% under continuous planting conditions and 76.6 and 53.3% under natural seeding deficiency conditions, respectively.

## Introduction

Cabbage is rich in nutrients, contains a variety of vitamins and mineral elements and has become one of the vegetables with the largest cultivated area in the world. In 2020, the cabbage total output of China was 339 million tons, which accounted for 47.71% of the global output, and it ranked first in the world. During the growth period of cabbage, pesticides need to be sprayed several times to resist the threat of diseases and insect pests. At present, the widely used continuous pesticide spraying method may cause soil and groundwater pollution due to the excessive use of pesticides (Zhao et al., 2016; He, 2020). However, automatic target pesticide spraying technology can be used to obtain target information online and spray pesticides at a specific site according to target positioning to reduce pesticide pollution in nontarget areas for environmental reasons, allowing wide application prospects. There are two ways to conduct target pesticide spraying for field cabbage. The first approach is to spray pesticides to prevent diseases and insect pests of cabbage, that is, the cabbage crop is the target of pesticide spraying, and the other approach is to spray the weeds that damage cabbage fields, that is, the weeds associated with cabbage are the target of pesticide spraying. The keys to realizing automatic target pesticide spraying are the online classification and identification of cabbage and weeds.

Machine vision technology has the advantage of being fast and accurate, and is most widely used in crop and weed classification and recognition research (Wang et al., 2019). The technique focuses on crop/weed classification by features such as colour, texture, and shape (Woebbecke et al., 1995), then the traditional neural network or deep learning algorithm is used to classify crops/weeds. Field environments are complex and variable unstructured environments, and strength of illumination and spectral content may change over time. Direct sunlight causes highlights and shadows in the field of view, which makes segmentation and feature extraction of vegetation (crops and weeds) from the background (bare soil, rocks and residues) difficult. Therefore, it is necessary to design systems and their algorithms that are robust to changing light for the design. In the algorithm, in order to minimize the effect of light on segmentation, researchers have tried colour space transformations (García-Mateos et al., 2015; Tang et al., 2016), but the colour component channels of space are obtained by nonlinear transformations of R, G, and B components, which are prone to local noise in edge regions with significant colour mutations and slow computing time (Yuan et al., 2020). Some researchers have directly investigated the accuracy of relevant algorithms for recognition of highlight and shadow problems under natural lighting conditions. Zheng combines mean shift segmentation algorithm and BP neural network to realize the segmentation of background and green plants, but this method is time-consuming (Zheng et al., 2017). Based on the assumption that the colour is gradually changing between the highlight region and the adjacent non-highlight region, Ye uses probabilistic superpixel Markov random fields to resist the strong illumination for the effect of shadows on segmentation under strong illumination conditions (Ye et al., 2015). The above methods are tested on the off-line images collected under natural light. No real-time identification test of field environment was conducted.

Multispectral cameras can provide several narrow spectral band vegetation features that are used as weed identification, avoiding light variation to some extent, but in order to make weed detection robust to light variation, white diffusers or the colour-checker white patch must be applied to estimate the strength of illumination (Shen et al., 2007; Macaire, 2021), which is a complex method for system model construction. Natural light is the most convenient for outdoor field work, but it is highly variable and not always reliable, and diffuse strip light sources with uniform intensity can eliminate many of the problems encountered with natural light Brown and Noble (2005). Therefore, physical methods such as artificial lighting and shading, have been used in many studies to obtain constant light conditions. By adding a hood and artificial light source approach is a simple and effective way to cope with variable natural illumination greatly reduces the difficulty of developing image processing algorithms (Pulido-Rojas et al., 2016; Bawden et al., 2017; Hall et al., 2018), and improves the accuracy of target identification. Agricultural field robots use hoods and artificial lighting to control recognition area illumination and improve recognition accuracy (Chebrolu et al., 2017; Elstone et al., 2020). Therefore, the lighting method with active light source under the hood has better implementation in target recognition. This paper uses the active light source method under the hood for target recognition.

Diverse and irregular weeds are distributed among crops and need to be identified using appropriate classifiers, which mainly include traditional machine learning-based classification and deep learning-based classification. Deep learning has a unique network feature structure that allows for higher-level features by learning local features from the bottom and then synthesizing these features from the top. However, current deep learning methods rely on large datasets for training (Kounalakis et al., 2019). Deep learning models need to be deployed to embedded mobile devices for practical applications, but embedded devices have low arithmetic power and are expensive, and current deep learning-based target recognition is mostly in the model construction and optimization stage, with few practical deployment applications [15]. With the help of traditional machine learning, such as artificial neural network (ANN; Bakhshipour et al., 2017; Bakhshipour and Jafari, 2018), k-NN clustering (Kazmi et al., 2015), support vector machine (SVM; Ahmed et al., 2012; Chen et al., 2020) and random forest (RF; Lottes et al., 2016), researchers have combined different types of features to achieve the classification of crops and weeds. Among them, SVM is one of the classical supervised machine learning methods, which is able to construct the maximum classification surface between different classes with strong generalization ability (Azarmdel et al., 2020), have high accuracy and efficiency in the case of small sample size and nonlinearity, and is the most commonly used method to distinguish between crops and weeds (Chang et al., 2021).

Identification speed is another important factor for the application of the algorithm in the field. In the process of field target identification based on an SVM, Mahajan et al. proposed an SVM-based plant species identification model and selected seven features of leaves as the input of the SVM. García-Santillán and Pajares (2018) proposed a classification and identification approach for weeds among crops based on Bayes’ minimum criterion for the Mahalanobis distance and obtained an accuracy of 91.8% and a processing speed of 280 ms/f. Raja et al. (2020) designed a kind of micro herbicide spraying system for weed control in vegetable fields, in which two cameras and an on-board computer connected *via* an Ethernet port (Intel core i7, 3.4 GHz, 12 GB DDR3 RAM) were used for image acquisition and processing, with a processing time of 160 ms/f. Esau et al. (2013) designed a variable pesticide spraying control system based on vision. According to the test results in a wild blueberry field, the image acquisition and processing time of the system was 0.079 ms, but the system only used green information to distinguish bare soil from wild blueberries. In this way, although it has a simple algorithm, it is quite different from target identification in a field of view. Wang et al. (2021) realized row segmentation for corn roots and stubble based on an SVM and established a feature vector by selecting 21 features from the colour and texture features of the extracted target and background as the input of the SVM identification model; an average model identification time of 60 ms was obtained. The above identification process based on an SVM involves a large number of features; however, for real-time target pesticide spraying at high speeds, as the number of features of the SVM affects the processing time of the SVM, the long image processing time of complex model will inevitably affect the spraying accuracy. This study uses cabbage as the recognition target, and the construction features of the support vector machine model are preferred to improve the speed of the algorithm according to the difference of shape features between cabbage and weeds. And the accuracy of the algorithm and the target application control system was verified by building a target spraying system and field trials, and a quantitative evaluation was given. This study is expected to improve the accuracy of on-target application, improve the intelligence of on-target application for field vegetables, and effectively reduce pesticide use and environmental pollution. This study is expected to improve the accuracy of target pesticide spraying, enhance the intelligence level of target pesticide spraying of vegetables in a field, and effectively reduce the environmental pollution caused by the use of pesticides.

## Materials and Methods

### Overall Design of the Target Pesticide Spraying System

**Figure 1:**
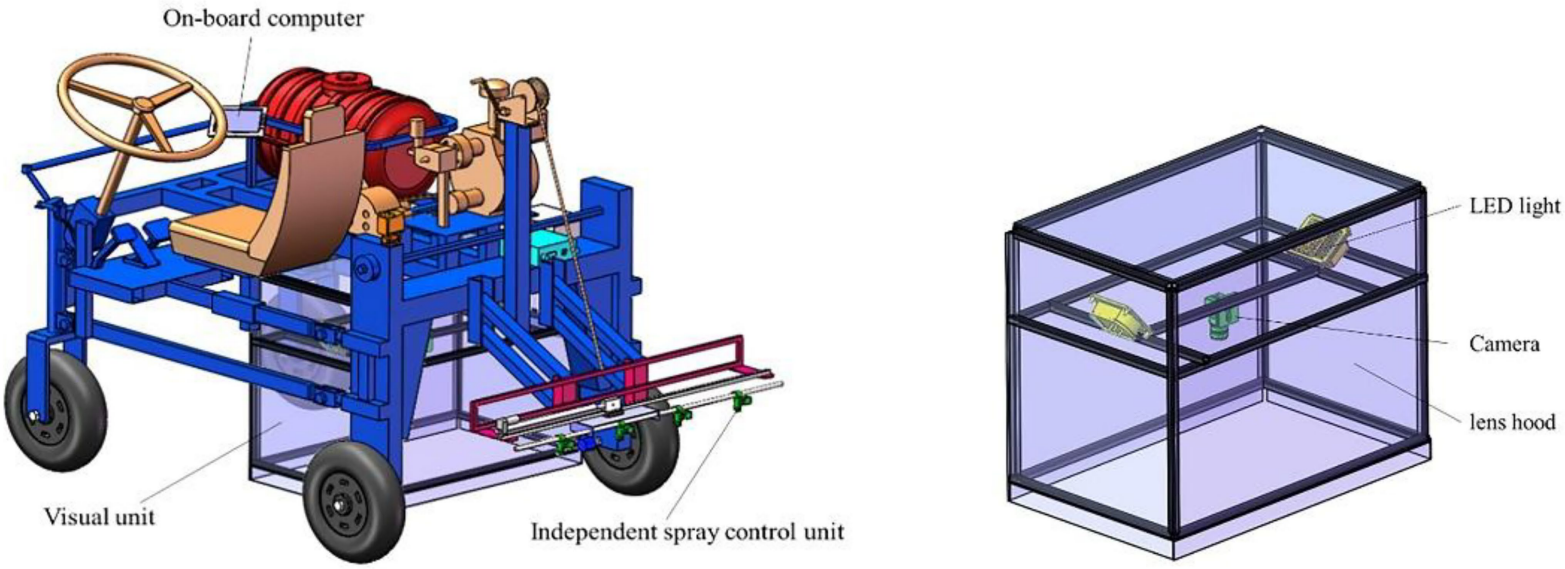
Components of the target identification and pesticide spraying control system based on an active light source.

The target identification and pesticide spraying control system for cabbage crops based on an active light source is mainly composed of a visual unit and independent spray control unit, as shown in Figure 1. The visual unit primarily consists of an on-board computer (SKU-4, Shenzhen Dehang Intelligent Technology Co., LTD.), a camera (MER-500-14U3C, Beijing Image Vision Technology Branch, China Daheng (Group) Co., LTD.), an LED light source (HL-BL68738R, HaoLi Automation Technology Co., LTD.), and a light shield. The independent spray control unit is mainly made up of an electronic control unit (ECU; C37, Suzhou Hesheng Microelectronics Technology Co., LTD.), a self-developed solenoid valve, a spraying body (Vp110-02, Ningbo Licheng Agricultural Spraying Technology Co., LTD.), a pressure sensor (QDW90A-RG, Huaibei Ruigan Electronic Technology Co., LTD.), a flow sensor (YF-B2/B4, Saibao Electronic Technology Co., LTD.), and a vehicle speed sensor.

**Figure 2:**
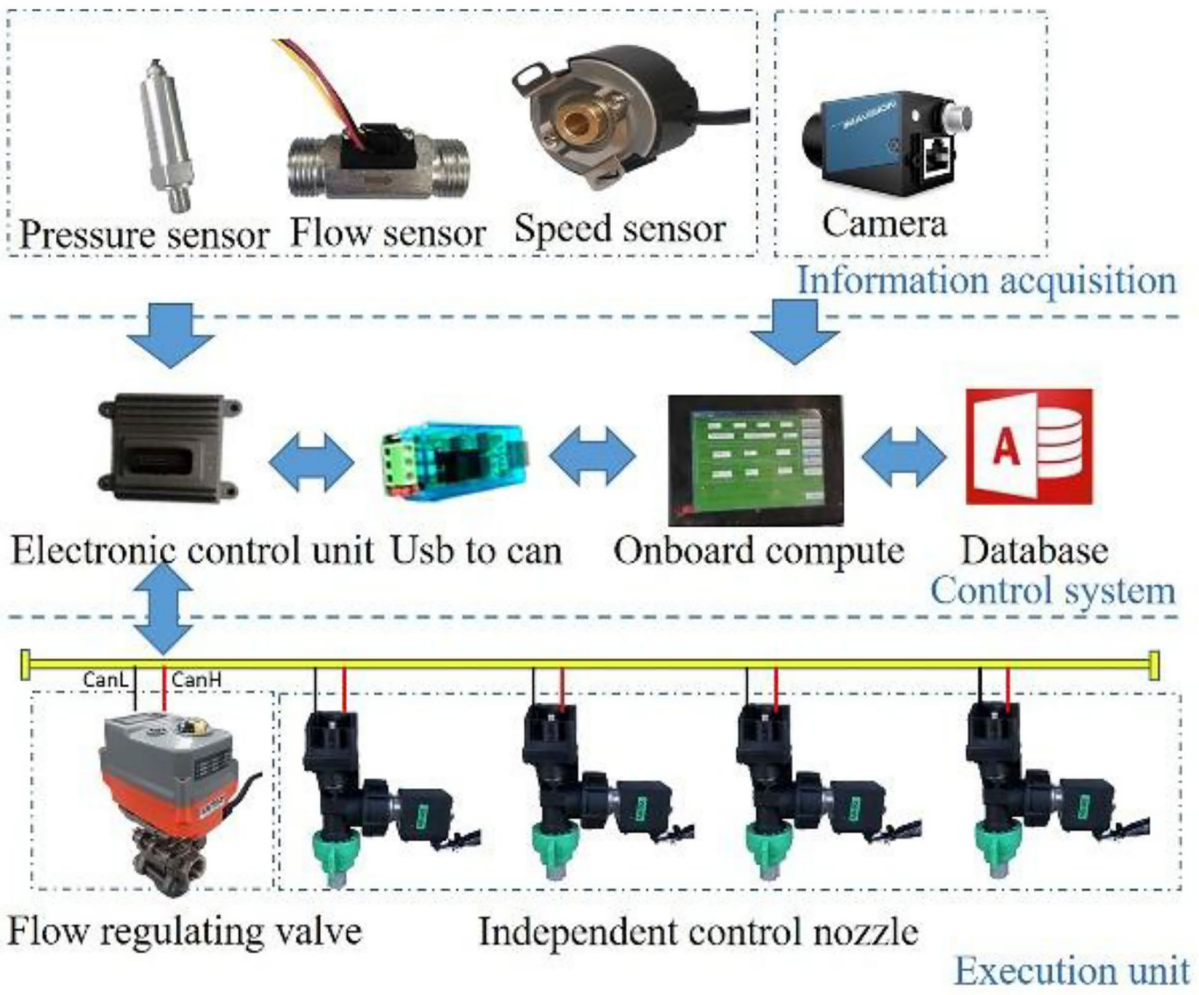
Components of the target variable spraying system based on vision.

When working, the sprayer starts to move forward, and the camera collects field images in real time and transmits them to the on-board computer, which identifies the target in real time by processing the algorithm and issues control commands to the ECU through the USB-CAN module. Then, the ECU obtains the current speed in real time through the speed sensor and opens or closes the solenoid valve installed on the resistance drop valve to realize opening and closing control of the nozzle according to the target position information, distance information between the nozzle and the camera, the computing and processing times and the transmission delay time received to achieve precise target spraying. For the pressure fluctuation caused by different numbers of nozzles or possible changes in the walking speed during the target pesticide spraying process, the system would automatically adjust the opening and closing size of the electric flow regulating valve to make the pressure stable within a set range and ensure the implementation of target pesticide spraying. Moreover, nozzles are distributed on the spraying rod, and the water inlet of the spraying rod is equipped with a flow sensor to monitor the spraying amount in real time. The composition of the control system is shown in Figure 2.

### Classification and Identification Method of Cabbage and Weeds Based on an SVM

#### Image Acquisition

**Figure 3:**
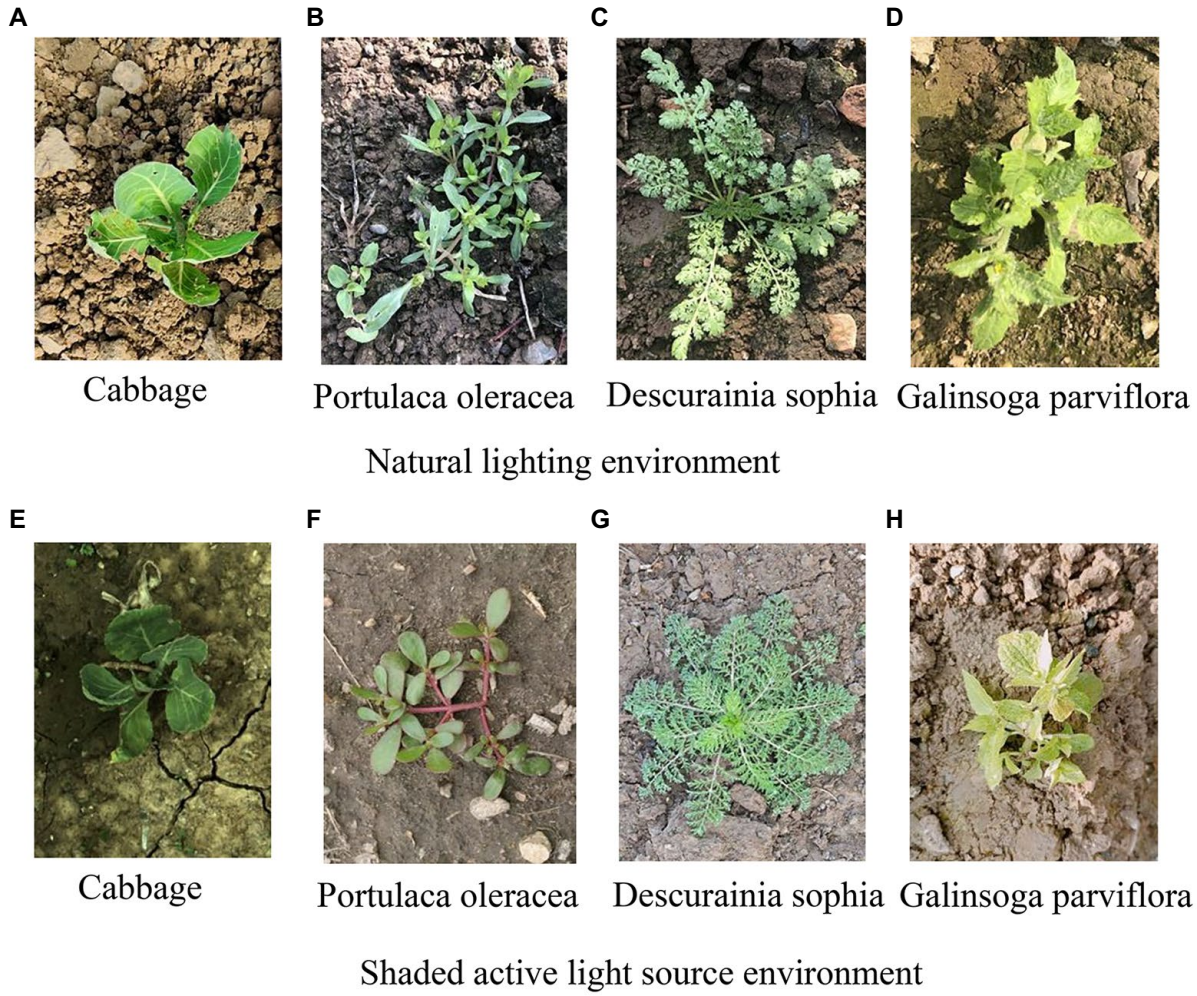
Partial acquisition samples. **(A)** Cabbage. **(B)** *Portulaca oleracea*. **(C)** *Descurainia sophia*. **(D)** *Galinsoga parviflora*. Natural lighting environment. **(E)** Cabbage. **(F)** *P. oleracea*. **(G)** *D. sophia*. **(H)** *G. parviflora*. Shaded active light source environment.

The collection objects include cabbage in the early growth stage and the three most common weeds, *Portulaca oleracea*, *Descurainia sophia*, and *Galinsoga parviflora*, which are in the field at the same time. From August 5, 2020 to August 30, 2020, from April 15, 2021 to May 12, 2020, and from July 26, 2021 to August 22, 2021, image acquisition was carried out by a GoPro digital camera (HERO_9, GoPro Co., LTD.) and Daheng Mercury series camera (MER-500-14U3CMER-U3C, Beijing Image Vision Technology Branch, China Daheng Co., LTD.) at the Beijing Xiaotangshan National Precision Agriculture Demonstration Base. The GoPro digital camera has good anti-shake capability, and the acquisition was carried out with the lens plane mounted parallel to the ground on the test sprayer with a height of 0.8 m above the ground and a field of view size of 1.55 m^*^0.76 m. In order to improve the adaptability of the algorithm to illumination, the acquisition is divided into natural illumination environment and shading active light source environment, 100 images of cabbage and 100 images of each species weed in the two light source environments were randomly selected. Mer-500-14u3c camera has a resolution of 2,592 pixels × 1,944 pixels, installed on the Pan tilt of the camera tripod, and the camera mounting height was 0.5 m. Adjust the angle between the camera lens and the ground through the pan tilt to 0°, 10° and 20°, respectively, and 100 images of cabbage and 100 images of each species weed were collected at each angle. To ensure the diversity of the image samples, the acquisition was carried out at different surface water content, and the surface images would present different colour and texture information, and some of the samples are shown in Figure 3. The experiment randomly selected 400 images of cabbage and various weeds, respectively, a total of 1,600 images as training samples, and the remaining 400 images as test samples. In order to improve the training speed and recognition accuracy of the recognition model, as well as the subsequent segmentation performance (Yang et al., 2018), the acquired image size is first Gaussian transformed by the OpenCv library pyrDown() function on the original image, and then by discarding the even rows and even columns, realize downsampling to adjust the image to 640 pixels × 480 pixels.

#### Identification Model Training on Cabbage Images Based on SVM

**Figure 4:**
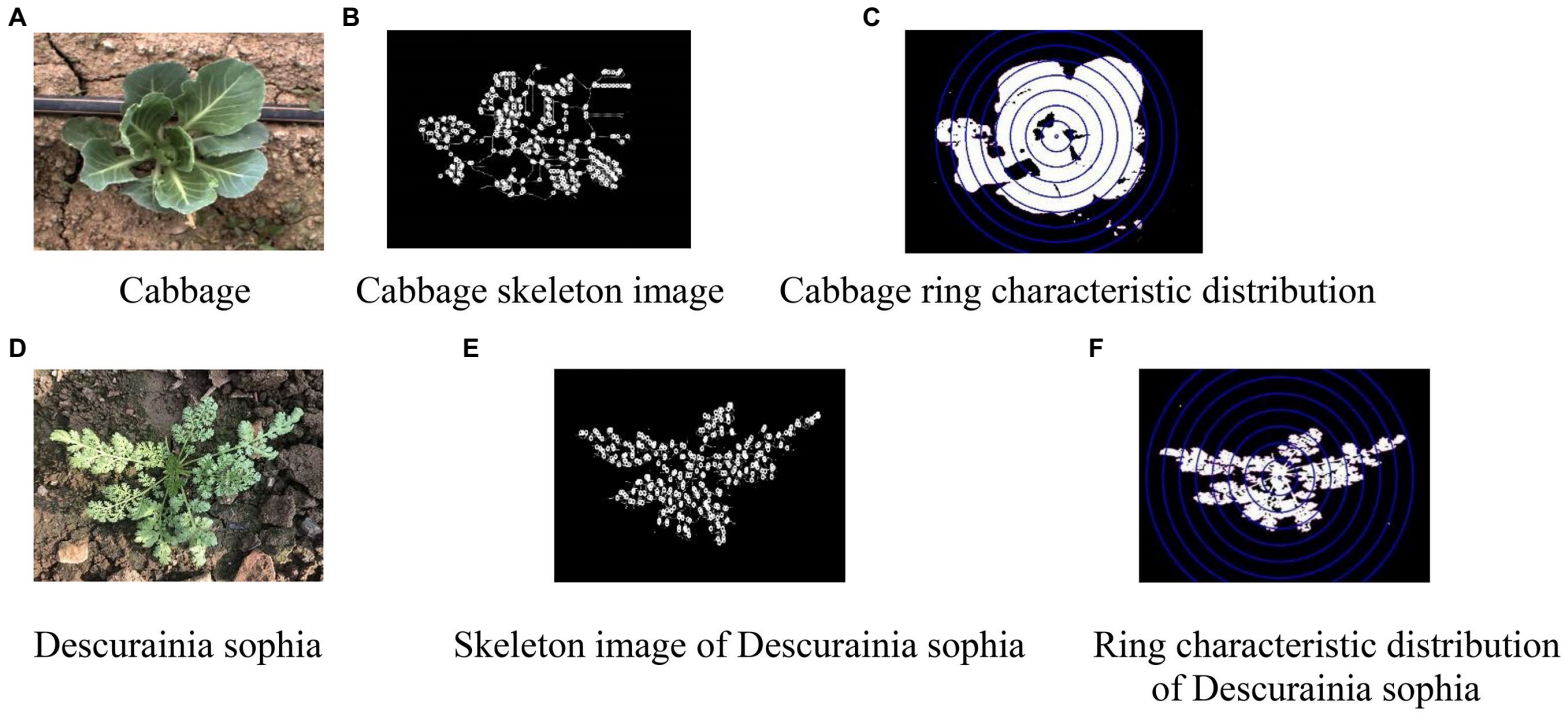
Characteristics of concentric rings. **(A)** Cabbage. **(B)** Cabbage skeleton image. **(C)** Cabbage ring characteristic distribution. **(D)** *Descurainia sophia*. **(E)** Skeleton image of *D. sophia*. **(F)** Ring characteristic distribution of *D. sophia*.

First, the green information in the background was extracted by the ultragreen operator (2G-R-B), average filter core size is 5^*^5 pixels. Then, noise points were removed by means of average filtering, and finally, an open and close operation was used to eliminate small objects, the size of corrosion and expansion structure elements in opening and closing operation is 3^*^3 pixels, smooth the boundary of large objects, and fill small cavities in the body. When used shape features are used in the classifier, the model has a better computing speed compared to when the same amount of texture features are used in the classifier; hence, because cabbage is the identification target, on the basis of image segmentation, this paper selected the circularity degree of Rod and the maximum inscribed circle radius R as classification features and proposed a point-to-line ratio and concentric ring structure according to the different shape features of cabbage and weeds. Of which, the point-to-line ratio refers to the ratio of the number of intersection points of the skeleton line to the length of the skeleton line after skeleton extraction from images, which was denoted as RAT_I_L. Taking cabbage and *D. sophia* as an example, the original images of cabbage and weeds are shown in Figures 4A,D, the extracted skeleton lines and intersection points are shown in Figures 4B,E, and the distribution characteristics of the concentric circles are shown in Figures 4C,F. Then, with the centre of mass as the centre of the circle and k^*^r/8 (*k* = 1,2… *N*) as the radius of the circle, the radius, centre of mass of the minimum enclosing circle, and the proportion of white pixels within each ring were calculated. Furthermore, the proportion of white pixels within each ring was subjected to quadratic curve fitting for the extraction of curve coefficients *a* and *b*, and the fitted curve coefficient ratio b/a was taken as the construction feature. Taking into consideration the different proportions of rings and the sizes of crops and weeds, in this paper, *n* = 8 was selected.

The training objective of the cabbage identification model based on an SVM is to find the hyperplane that can divide cabbage and weed samples with the largest interval. The reasonable selection of the kernel function type and parameters has a significant influence on the performance of the identification model. After many experiments, the radial bias function (RBF), which has high identification accuracy, was selected as the kernel function of the SVM identification model for cabbage.


(1) $$K\left(x_{i},x\right)=\exp\left(\gamma ∥x_{i}-x{∥}^{2}\right)$$

where $\gamma =\frac{1}{2\sigma }$ .

The parameters that affect kernel function include penalty factor *C* and kernel function *σ* (He and Luo, 2019), the penalty factor *C* is the control of the penalty degree of the misclassified samples. The larger the penalty is, the heavier the penalty is, but its generalization ability will also be reduced at the same time. *σ* is width parameter of the kernel function, indicating the control over the radial range. Penalty factor *C* and kernel function *σ*. The selection of SVM classifier is very important. The optimal values of penalty coefficients *C* and *σ* were obtained by N-fold hierarchical cross verification based on the grid search method. According to related literature, *n* = 5 was selected in Equation (1) (Jung, 2017), where n represents the amount of data and d represents the number of features.


(2) $$\left\{ \begin{array}{c} N\approx \log\left(n\right)\\ n/N>3d \end{array}\right.$$

Then, the selected ranges of *C* and *σ* were 2^−15^ ~ 2^15^ and 2^−10^ ~ 2^10^, respectively, and the optimal *C* and *γ* were selected as the optimal parameters after the search was conducted in the plane composed of *C* and *γ*. The maximum average accuracy of hierarchical cross verification of the training set was taken as the index. To select the optimal classification feature combination among b/a, RAT_I_L, ROD and R, the four features were normalized and trained through the SVM method based on the radial kernel function.

The training platform was a Hewlett-Packard (HP) computer, the processor was an Intel (R) Core (TM) i7-9750H, 12-core 2.60 GHz, 32 GB RAM, the graphics card was an NVIDIA GeForce GTX 1650, and the operating system was Windows 10. The visual open-source libraries used in the training stage of image processing and SVM-based cabbage contained OpenCV and SVMlight.

In order to verify the feasibility of the method in this paper and its classification performance, the system is tested using a designed test set of samples. Back Propagation Neural Network (BPNN) and Random Forest (RF) were selected to train the cabbage/weed recognition model, which constitutes the BPNN classification method and RF classification method, and compared with the support vector machine method used in this paper. Among them, after referring to the settings of the main parameters of BP neural network and RF classification method in machine learning, image processing and other related studies (Goel et al., 2003; Wu et al., 2013), it was determined on the basis of the preliminary experiments that the BP neural network in this study adopts a 4500-300-20-2 4-layer structure, the activation function is a sigmoid function, the learning rate is 0.02, the target error is 0.01.The random forest algorithm is operated in the Pycharm environment, and the Random Forest Classifier classification module is called, through sklearn ensemble. The number of decision trees (n_estimators) is 100, the maximum depth of decision tree (max_depth) is 400, the minimum number of split samples (i.e., the minimum number of samples required to split the nodes of the decision tree) min_samples_split is 2, the minimum number of leaf node samples (i.e., the minimum number of samples required to be included in a leaf node) min min_samples_leaf is 1, and max_features (i.e., the number of feature variables to be considered in finding the best node split) is $\sqrt{N}$ , *N* is the number of features. In order to objectively evaluate the performance of the proposed methods in this paper, the accuracy, precision, recall, and computing time are selected to quantitatively evaluate the recognition results of different methods.


(3) $$\mathrm{Accuracy}=\frac{TP+TN}{TP+FP+FN+TN}\times 100\%$$

(4) $$\mathrm{Precision}=\frac{TP}{TP+FP}\times 100\%$$

(5) $$\mathrm{Recall}=\frac{TP}{TP+FN}\times 100\%$$

Where TP is True positive; FP is False positive; TN is True negative; FN is False negative.

### Target Positioning Method

**Figure 5:**
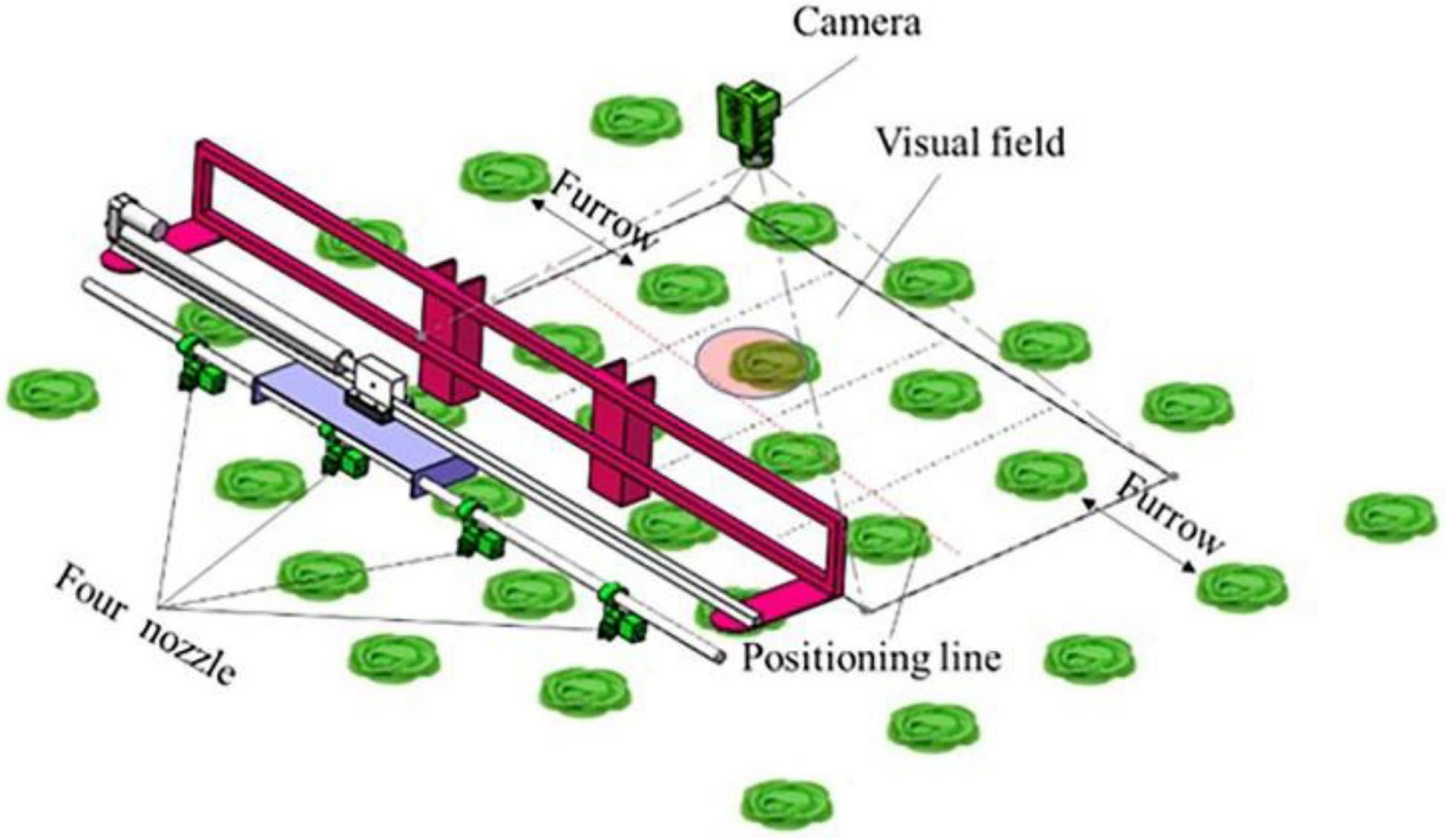
Schematic diagram of spraying control with multiple targets in a single field of view.

The cabbage planting agronomy is shown in Figure 5, where every 4 rows of cabbage was a ridge, with a ridge spacing of 50 cm, row spacing of 35 cm, and plant spacing of 40 cm. When operated, the operation range of the spraying machine reached 50 cm, so that 4 rows of cabbage in a ridge were identified at the same time. In addition, each row of cabbage was divided into an identification area to install a nozzle accordingly to ensure that each nozzle corresponded to a row of cabbage. The system controls the opening and closing of the corresponding solenoid valve according to the cabbage target information to realize targeted application. Additionally, the camera adopts a zoom lens with a mounting height of 0.9 m and a field of view width of 1.5 m, and the clarity of the image is controlled by adjusting the focal length. Because the actual camera imaging pixel length and width is 1,280^*^1,024, the length of the field of view in the advancing direction is 120 cm.

**Figure 6:**
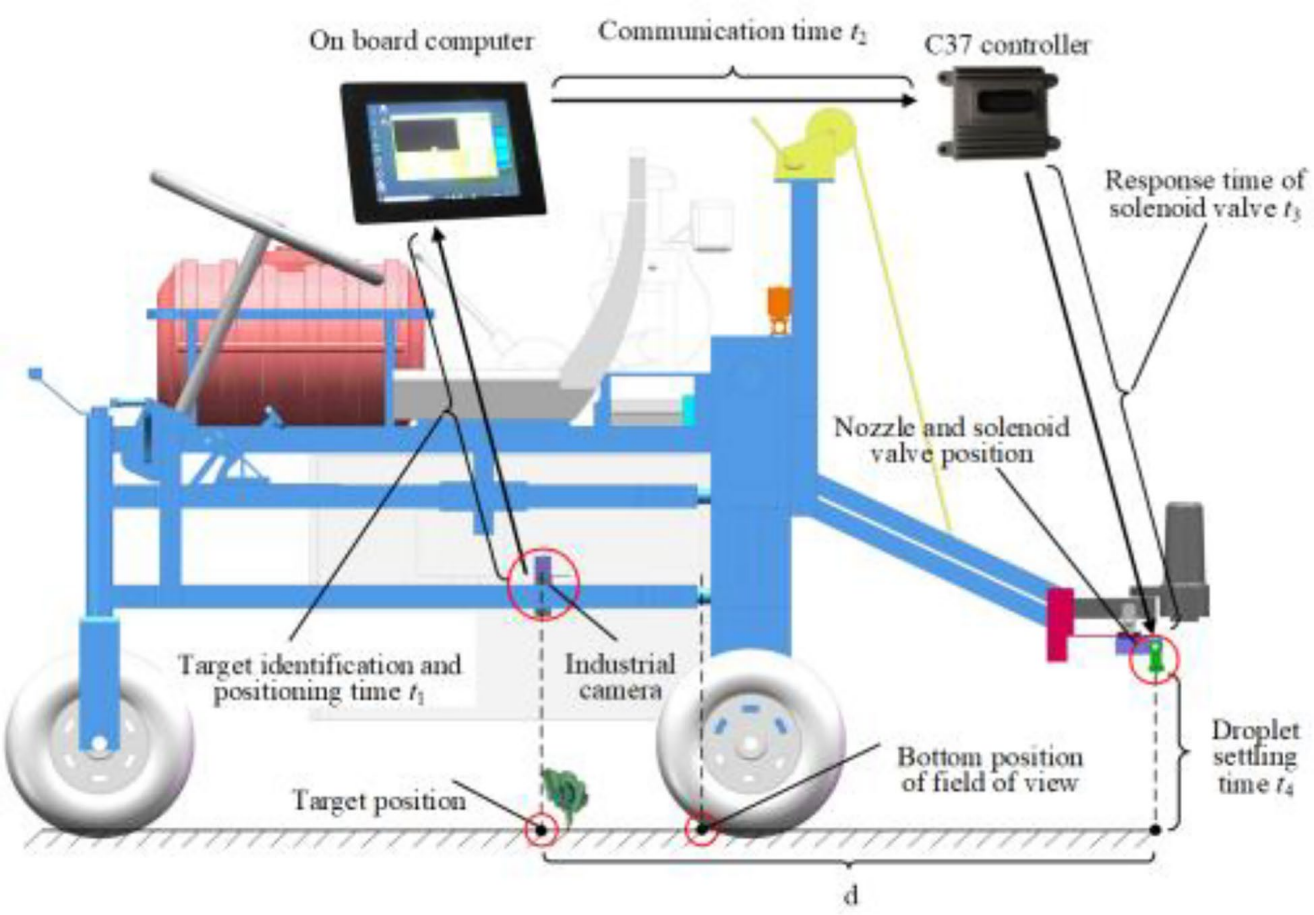
Schematic diagram of each delay component of control system.


To position the target accurately during the walking process of the spraying machine, it is necessary to obtain the target position and spraying machine walking distance. The walking distance is obtained by the encoder installed on the walking wheel. When forward distance is equal to the longitudinal length of the field of view, the camera is triggered to take photos, and the target can be positioned in the field of view García-Santillán and Pajares (2018). However, the skidded walking wheel and uneven ground caused a large cumulative error, resulting in a decrease in the quality of target positioning. This study used video streaming to locate the target and establish the region of interest in the field of view, and the longitudinal positions of the targets (Yn) before and after the frame image was calculated (n is the number of image frames, where *n* = 1,2,3… *N*). When equation 3 is satisfied, it was judged that a target was passed by, as shown in Figure 6.

(6) $$\{\frac{Yn>Y\;\mathrm{threshold}}{Y_{n-1}<=Y\;\mathrm{threshold}}$$

Additionally, there is a certain distance *d* between the camera target and the nozzle and a running time of the control system, which includes target identification and operation time *t*_1_, communication time *t*_2_, solenoid valve response time *t*_3_, and fog droplet falling time *t*_4._ The time delay model of target control is shown in Figure 6. To be specific, the graph acquisition and processing time was obtained through the gettickcount() function; by Yuan’s method, the time was 38 ms, and the response time of the solenoid valve was 20 ms (Yuan et al., 2020). According to the high-speed photography test, a 115,200 baud rate and binary transmission was applied under the conditions that the system pressure was 0.42 mp and the spraying height was 0.35 m. The fog droplet falling time and communication time between the upper computer and the lower computer controller were 35 ms and 820 μs, respectively. Based on these results, it can be calculated that from system image acquisition to solenoid valve response, there was a total of 138.82 ms. Then, according to Figure 6, when the distance between the lower edge of the region of interest and the nozzle is 0.7 m. To achieve accurate target pesticide spraying, the following conditions should be met:


(7) $$T=\frac{L-\left(t_{1}+t_{2}+t_{3}+t_{4}\right)*v}{v}$$

where *T* is the set delay time by the system, and the unit is seconds.

### Program Design of the Electronic Control System

The ECU receives the spraying instructions issued from the controller in real time through the CAN bus, controls the opening and stopping of the nozzle through the solenoid valve, and receives and processes real-time data, such as solenoid valve control data and the speed and pressure state of the pesticide supply system, to achieve accurate spraying control. Referring to the ISO11783 standard, the arbitration field and data field of the CAN bus protocol are designed. Specifically, the CAN packet includes the frame information (one byte), frame ID (four bytes), and frame data (eight bytes), and during data transmission, SC/SM, the start character of the frame, and the CRC check at the end of the frame were added. The data field protocol is shown in Table 1.

**Table 1:** Data field protocol of the pesticide spraying bus system.

Node	Identifier	Effective data length	Frame data meaning
TTC32	18E96664	8	Data0: Open state 0×0A Close state 0×00; Data2-Data3: Speed; Data4-Data5: System pressure; Data6-Data7: Flow;
On-board computer	0 CE76468 0 CE76469 0 CE76470 0 CE76471	8	Data0, Data1: Opening and closing distance of the solenoid valve corresponding to the cabbage; Data2, Data3: Opening and closing distance of the solenoid valve corresponding to the cabbage; Data4, Data5: Opening and closing distance of the solenoid valve corresponding to the cabbage; Data6, Data7: Opening and closing distance of the solenoid valve corresponding to the cabbage;

According to the above target positioning method, to achieve precise position spraying, the system delay time needs to be set according to different speeds. In this regard, after target identification, the upper computer sends the information in four packets, with an interval of 2 ms for each packet, which is longer than the communication time of packets to prevent packet transmission conflicts. The encoder was installed on the right rear wheel so that the C37 controller could read the pulse frequency of the encoder and convert it into the advancing speed of the spraying machine, with the accumulated pulses as the advancing distance. The controller of the lower computer stored the target information of four rows cabbages into four arrays upon receiving it from the host computer, after which the current driving distance was stored into an array called Distance[]. Table 2 shows that the target information includes the current distance when the ECU receives the target information. When the spraying control condition meets Equation (5), the nozzle is opened; otherwise, it is closed, effectively preventing the error accumulation of the displacement sensor caused by the tractor wheel skid.

**Table 2:** Comparison of the training results of different training feature combinations.

Training feature	Kernel function parameter	Number of SVMs	Correct classification rate of training samples/%	Correct classification rate of test samples/%	Operation time of test identification/ms
Rod, R,	*C* = 2^9^, *σ* = 2^−6^	23	78.9	76.0	23
RAT_I_L, Rod	*C* = 2^9^, *σ* = 2^−6^	23	82.3	81.7	26
RAT_I_L, R	*C* = 2^9^, *σ* = 2^−6^	49	89.3	87.9	26
RAT_I_L, b/a	*C* = 2^9^, *σ* = 2^−6^	13	90.3	90.1	29
Rod, b/a	*C* = 2^9^, *σ* = 2^−6^	13	90.7	86.2	27
b/a, R	*C* = 2^9^, *σ* = 2^−6^	11	92.6	90.7	27
RAT_I_L, Rod, R	*C* = 2^13^, *σ* = 2^−8^	13	92.3	90.1	32
RAT_I_L, b/a, Rod	*C* = 2^13^, *σ* = 2^−8^	13	94.6	91.2	33
RAT_I_L, b/a, R	*C* = 2^13^, *σ* = 2^−8^	11	97.6	95.7	35
Rod, R, b/a	*C* = 2^13^, *σ* = 2^−8^	11	94.3	93.1	34
RAT_I_L, Rod, R, b/a	*C* = 2^13^, *σ* = 2^−8^	9	97.8	95.9	38


(8) $$\mathrm{distance}+\mathrm{Distance}{\left[i\right]}_{\mathrm{open}}<\mathrm{distance}\left[\right]<\mathrm{distance}+\mathrm{Distacne}{\left[i\right]}_{\mathrm{close}}$$

**Figure 7:**
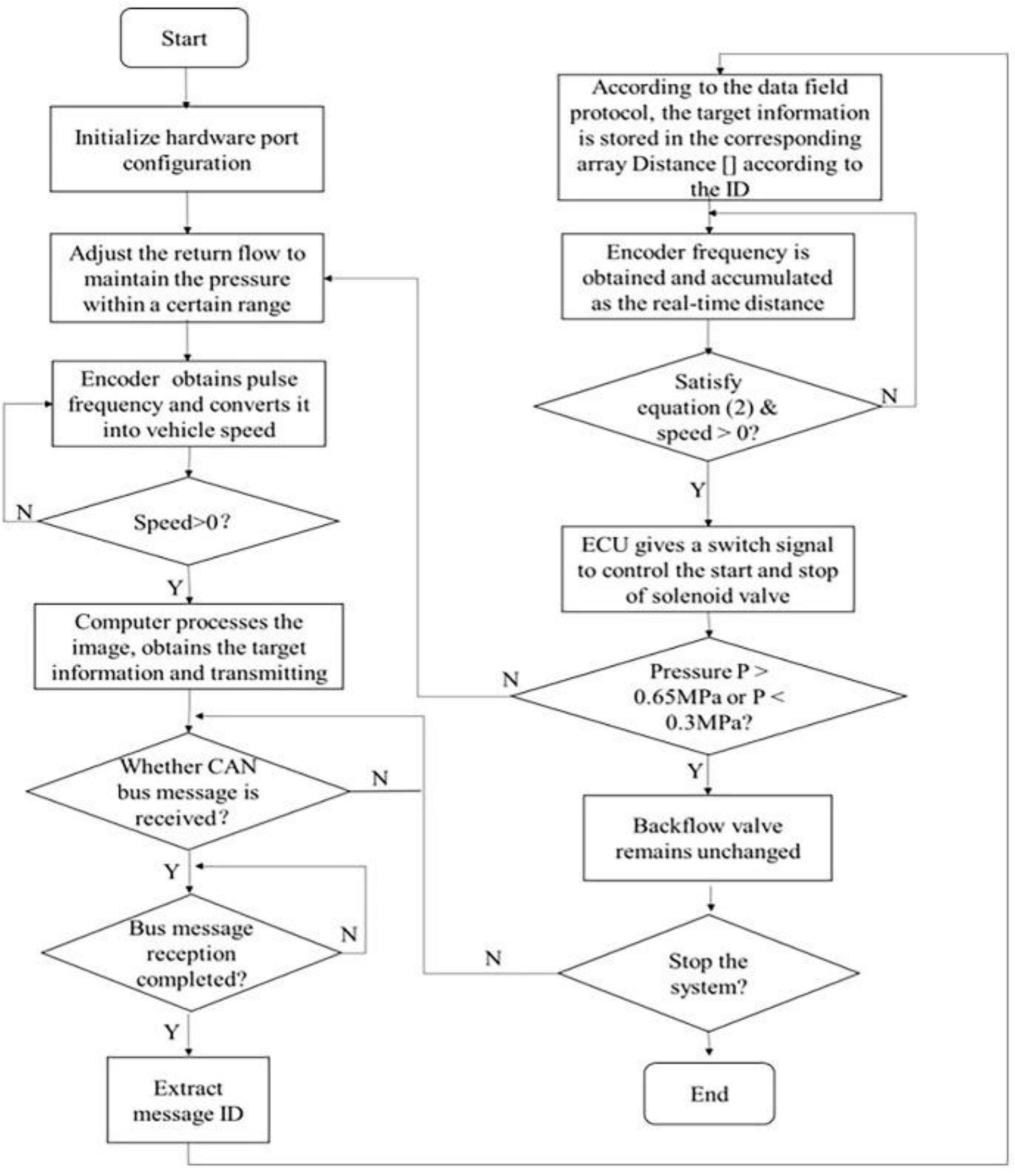
Control flow chart of targeted pesticide spraying.

The overall control flow chart is shown in Figure 7.

### Targeted Field Pesticide Spraying Test

**Figure 8:**
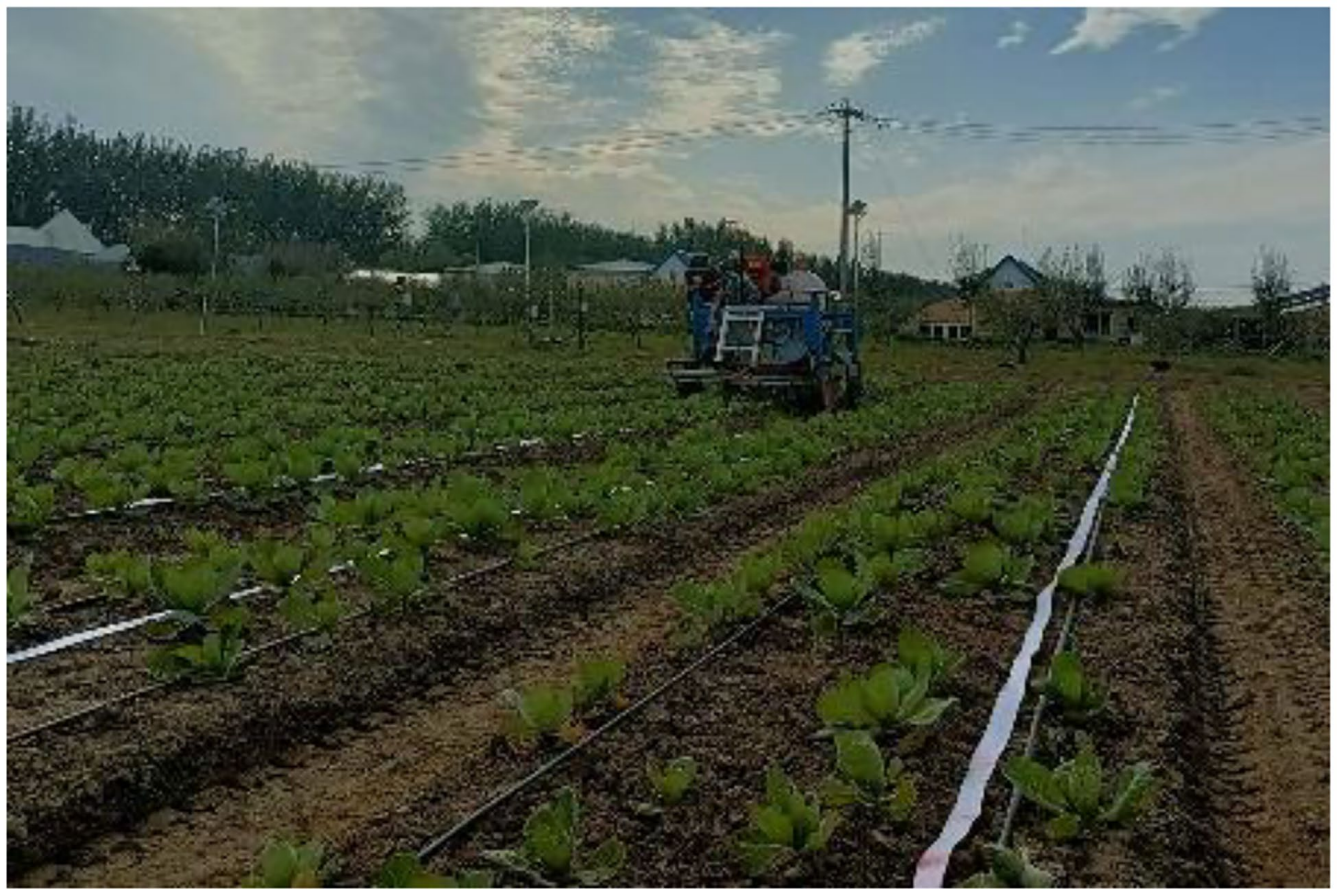
Field map of cabbage pesticide spraying.

**Figure 9:**
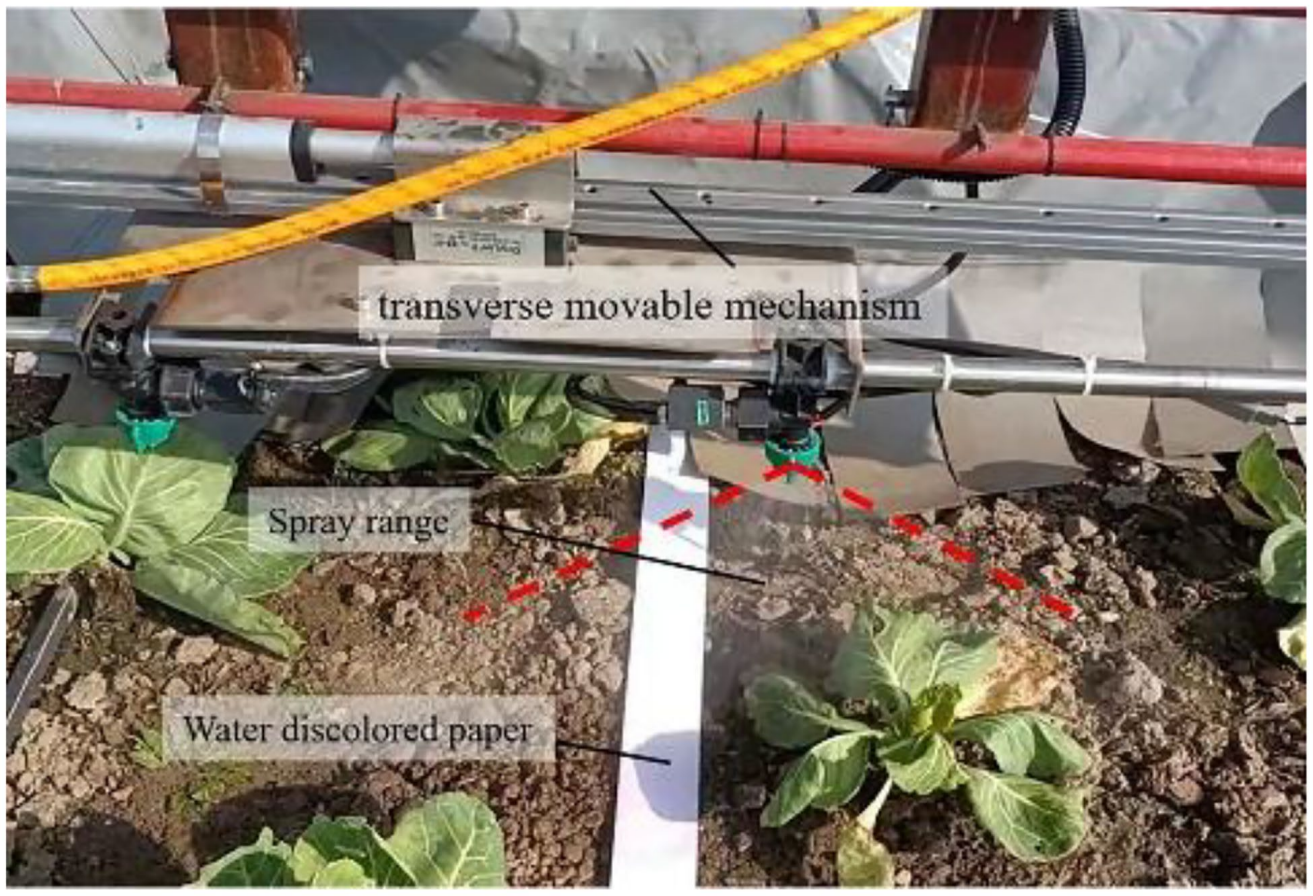
Layout map of a nozzle and paper strip.

To verify the accuracy and saving effect of the target pesticide spraying control system, experiments were carried out in the cabbage fields of Xiaotangshan National Precision Agriculture Research Demonstration Base, Changping District, Beijing. First, the measurement distance of the encoder and the shooting range of the image were calibrated, and the acquisition frequency was 28 frames/s. The row spacing of field cabbage was 35 cm, the plant spacing was 40 cm, and the ridge length was 70 m. Additionally, before the test, a white paper strip, which turned red when is encountered water, with a width of 40 mm was placed in the middle of the row of the cabbage. After spraying, the red colour deepened over time, but its depth did not affect the result discrimination. The test site is shown in Figure 8. By adjusting the movable mechanism, the spray body and the crop row deviated by 17.5 cm so that the paper strip was within the spray range. The overall layout is shown in Figure 9.

**Figure 10:**
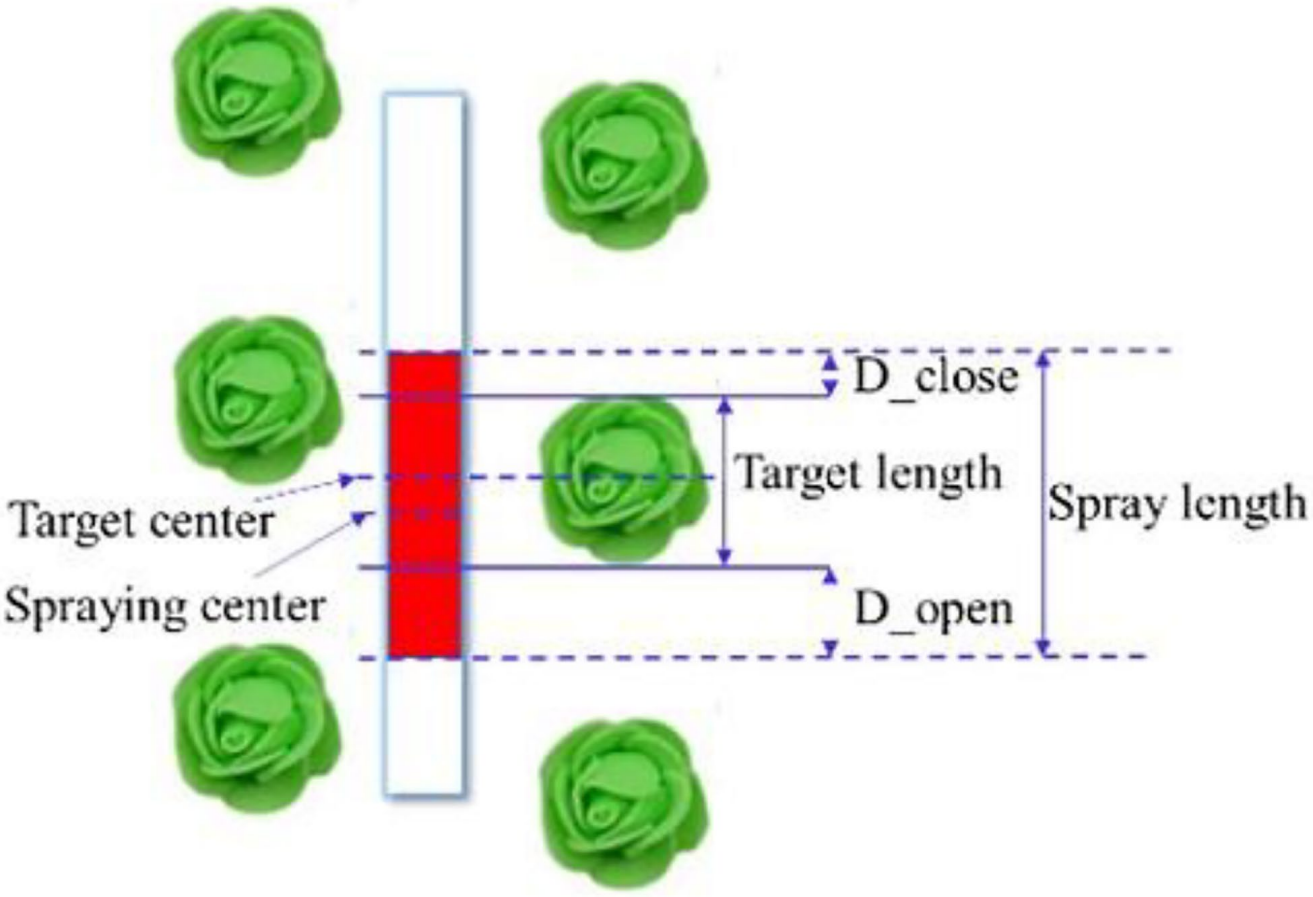
Effect definition diagram of the target spray.


To improve the contrast of the paper strip before and after spraying, the physical liquid was replaced by a solution composed of 0.1% crystal violet biological stain and distilled water. The length of the coloured area sprayed on the filter paper was measured and compared with the longitudinal length of the corresponding cabbage, as shown in Figure 10. The distances of the spraying range relative to the front end and back end of the advancing direction of the cabbage were called D_open and D_close, respectively, where D_open > 0 indicates the valve opened in advance, D_open < 0 indicates valve opening lagged, D_close > 0 indicates valve closing lagged, and D_close < 0 indicates the valve closed in advance. When the crops are contained in the coloured area, the target spray is considered to have hit the crops. When D_open < −7 cm or D_close < −7 cm (half of the average longitudinal length of the crop target), the spray is invalid; otherwise, it is effective. Again, the plant spacing range *L* is the plant spacing of transplanted cabbage, *l* is the length of cabbage in the advancing direction, and the ratio of the spraying length is the ratio of the target spraying length to the total spraying length under the effective spraying state. If the traditional nontarget pesticide spraying is the complete spraying within the range of the plant spacing, the theoretical saving rate ε is defined in Equation (6) under the condition of effective spraying. The average length of a cabbage is 160.3 mm, and the preset spraying advance distance and lag distance are both 30 mm; namely, the preset average spraying distance is 220.3 cm.

(9) $$\varepsilon =\left(L-D_{\mathrm{open}}-D_{\mathrm{close}}-1\right)/L^{*}100\%$$

On September 14, 2021, a hailstorm occurred in Changping District, resulting in crop seeding deficiency, with a seeding deficiency rate of 33% and a void rate of 76.6%. To obtain the pesticide saving rate, a targeted pesticide spray operation and a continuous spraying operation were carried out in a cabbage field with eight ridges under the condition of natural seeding deficiency and in a cabbage field with 10 consecutive cabbage under the condition of no seeding deficiency (the void ratio is 65%). During the test, four speed modes, including one-gear low speed, one-gear high speed, two-gear low speed and mixed speed of the above three, were used to spray two-ridge cabbage fields. Then, the number of accurate sprays of cabbage and weeds, the number of missed sprays and the number of invalid sprays were counted, the length, opening distance, and closing distance of each spraying target were measured, and the actual dosage was obtained by measuring the volume of physical liquid.

## Test Results and Discussion

### Identification Test Based on an SVM

The identification results of different training feature combinations are shown in Table 3.

**Table 3:** Identification results of cabbage and weed samples.

Recognition methods	Average precision/%	Precision%	Recall/%	Time consuming/s
BPNN	81.88	82.76	86.74	0.033
RF	90.23	88.67	54.33	0.056
SVM	95.7	93.72	92.35	0.038

It can be seen from the table that all the classifiers can classify cabbage and various weeds as expected, which proves that the SVM has good high-dimensional and nonlinear processing abilities. Among them, the identification accuracy of the Rod and R feature combination is the lowest, with a test accuracy of 77.0%. In terms of the combination of three or four features, when the kernel function parameter is *C* = 213 and *σ* = 2–8, the SVM performance is optimal. Moreover, the RAT_I_L, Rod, R, and B/A feature vector combined has the highest accuracy of 95.9%, and some cabbage and weeds are incorrectly classified. A target-oriented plant identification accuracy of more than 95% is widely accepted in most cases (Li et al., 2019).

**Figure 11:**
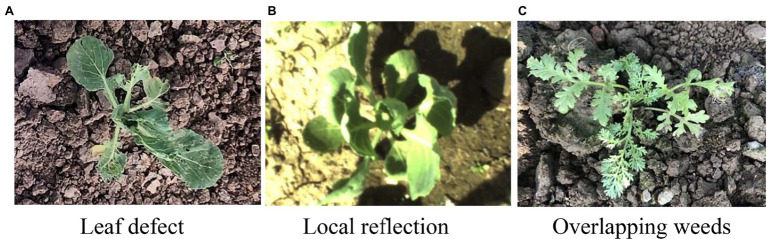
Partially incorrectly identified samples. **(A)** Leaf defect. **(B)** Local reflection. **(C)** Overlapping weeds.

The following reasons for sample identification errors are found through analysis. First, during the growth period of cabbage, natural disasters, such as hail, can cause serious damage to the leaves of some cabbage plants (Figure 11A), which can result in the gravity centre coordinate extracted from the images not being the centre of the plant, thus leading to an extraction error of sample shape features and a classification error. In addition, some cabbage samples have local reflections (Figure 11B), and some areas are not correctly segmented, thus leading to identification errors. Moreover, some samples of *D. sophia* overlapped with Shepherd’s purse samples and were wrongly identified as *P. oleracea*, which resulted in identification errors (Figure 11C). The running time is the main performance index of the target pesticide spray control system. By comprehensively considering the correct classification rate and test running time of samples, in this paper, a RAT_I_L, B/A and R feature vector combination was selected as the feature vector combination, with an average test time of 35 ms.

### Comparison and Analysis of Classification and Recognition Results by Different Models

Table 4 shows the average identification accuracy of the cabbage and weed BPNN model, RF model and support vector machine model, as well as the average precision, recall, and elapsed time of the different methods. The accuracy of BPNN model for cabbage and weeds is low (82.76%), which leads to serious misidentification, making the number of cabbage recognition as weed in the classification results was high, so the average accuracy rate was low (81.88%), and the average recall was high (86.74%); while the RF method misclassified too many weeds as cabbage, and its average identification accuracy (90.23%) was higher, while the average recall (54.33%) was lower; the average recognition accuracy, precision, and recall of the support vector machine were the highest among the three methods, 95.7, 93.72, and 92.35%, respectively.

**Table 4:** Comparison of classification results by different methods.

Name	Number	Number of correctly identified samples	Number of incorrectly identified samples	Identification accuracy/%
Cabbage	941	874	67	95.0
*Portulaca oleracea*	182	168	14	92.3
*Descurainia sophia*	266	256	10	96.2
*Galinsoga parviflora*	154	142	12	92.2

The average time of support vector machine algorithm is 0.038 s, which is 0.005 s more than the shortest BPNN segmentation method (0.033 s), but its accuracy, precision and recall rate are much higher than the other two methods, and the processing speed is at the millisecond level, which can better meet the requirements of real-time processing. In terms of segmentation performance and running time, this method can identify cabbage and different kinds of weeds from the complex natural environment, with strong robustness and high segmentation accuracy. Therefore, this paper selects the classification and recognition method of support vector machine.

### Field Target Spray Test

#### Accuracy of Target Identification

**Figure 12:**
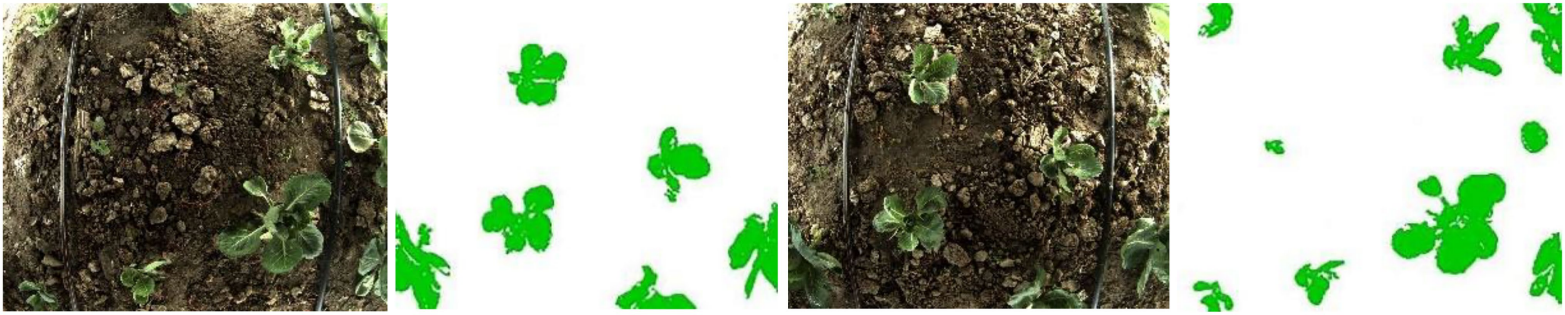
SVM identification results of cabbage in an active light source environment.

The SVM identification results of cabbage and weeds in an active light source environment are shown in Figure 12. In the figure, a partial cabbage sample in the boundary position of the field of view is not correctly identified in the case of local shooting, whereas the cabbage samples in nonboundary positions and weeds are wholly correctly identified.

**Figure 13:**
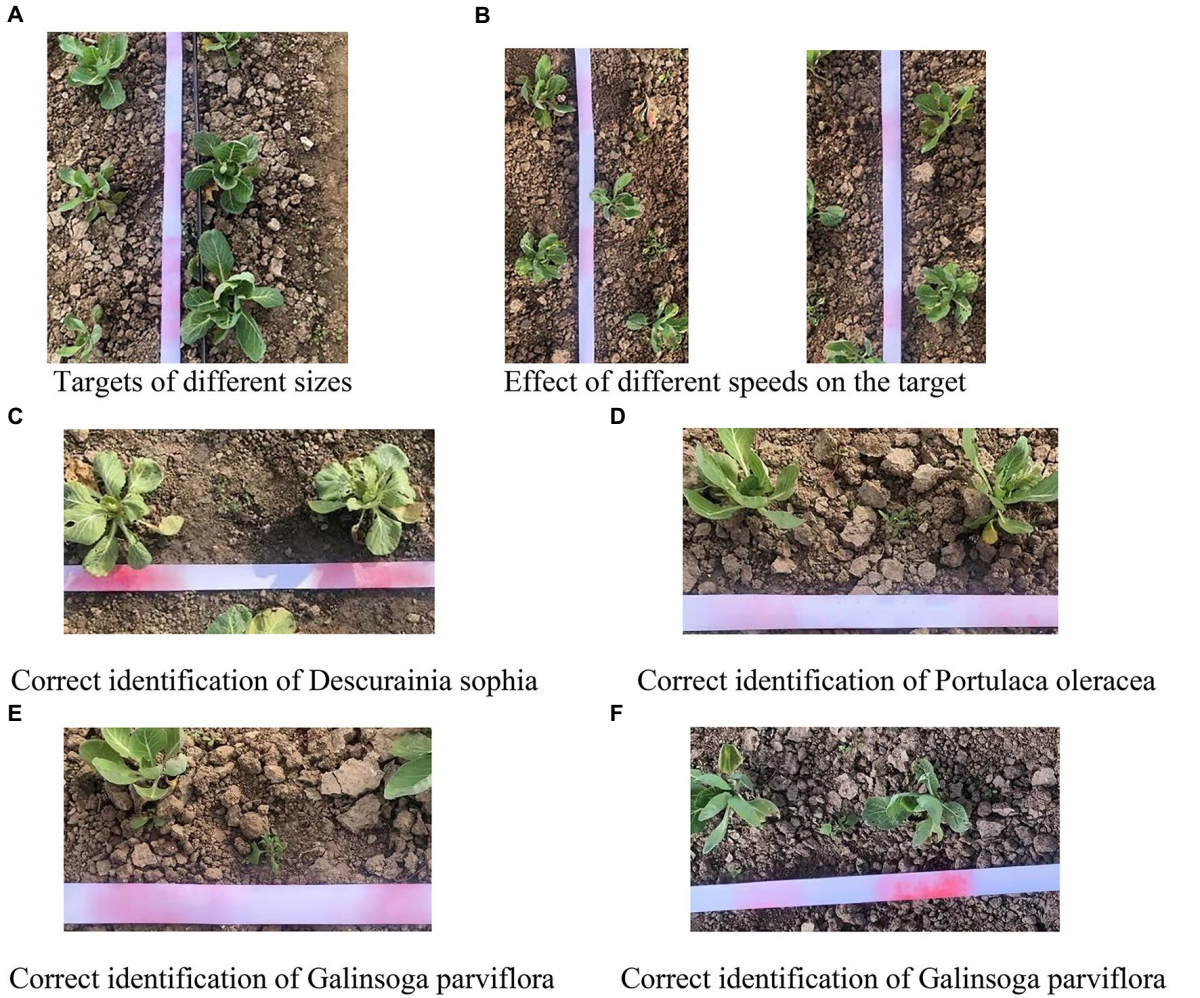
Correct identification of cabbage and various weed samples. **(A)** Targets of different sizes. **(B)** Effect of different speeds on the target. **(C)** Correct identification of *Descurainia sophia*. **(D)** Correct identification of *Portulaca oleracea*. **(E)** Correct identification of *Galinsoga parviflora*. **(F)** Correct identification of *G. parviflora*.

Table 5 shows the statistical spray results of cabbage and weeds, of which the identification accuracy of cabbage samples was 95.0%. Figure 13A shows the target spraying effect of cabbage plants of different sizes, from which it can be seen that the target control system can adjust the spraying distance according to the different sizes of the targets, indicating that the cabbage samples were correctly identified. Figure 13B shows the pesticide spraying of cabbage at different speeds, in which correct pesticide spraying is realized at both 0.52 and 0.93 m/s, indicating that the target is accurately identified at 0.52–0.93 m/s. However, the target offset at 0.93 m/s is larger than that at 0.52 m/s. Figures 13C–F shows the presence of weeds among the plants; all of the weeds were correctly identified and accurately sprayed. In addition, there is a certain difference in identification accuracy among different weeds; for example, *D. sophia* has the highest identification accuracy, and *G. parviflora* has the lowest identification accuracy, with an identification accuracy range of 92.2–96.2% and an average identification accuracy of 93.5%.

**Table 5:** Statistics of target spraying results at different speeds.

Speed /m·s^−1^	Number of targets	Number of ineffective sprays	Invalid spraying rate/%	Number of leakage targets	leakage rate/%	Number of effective spraying targets	Effective spraying rate/%
0.52	304	4	1.3	14	4.6	286	94.1
0.69	313	6	1.9	12	3.8	295	94.3
0.93	324	13	4.0	18	5.6	293	90.4

**Figure 14:**
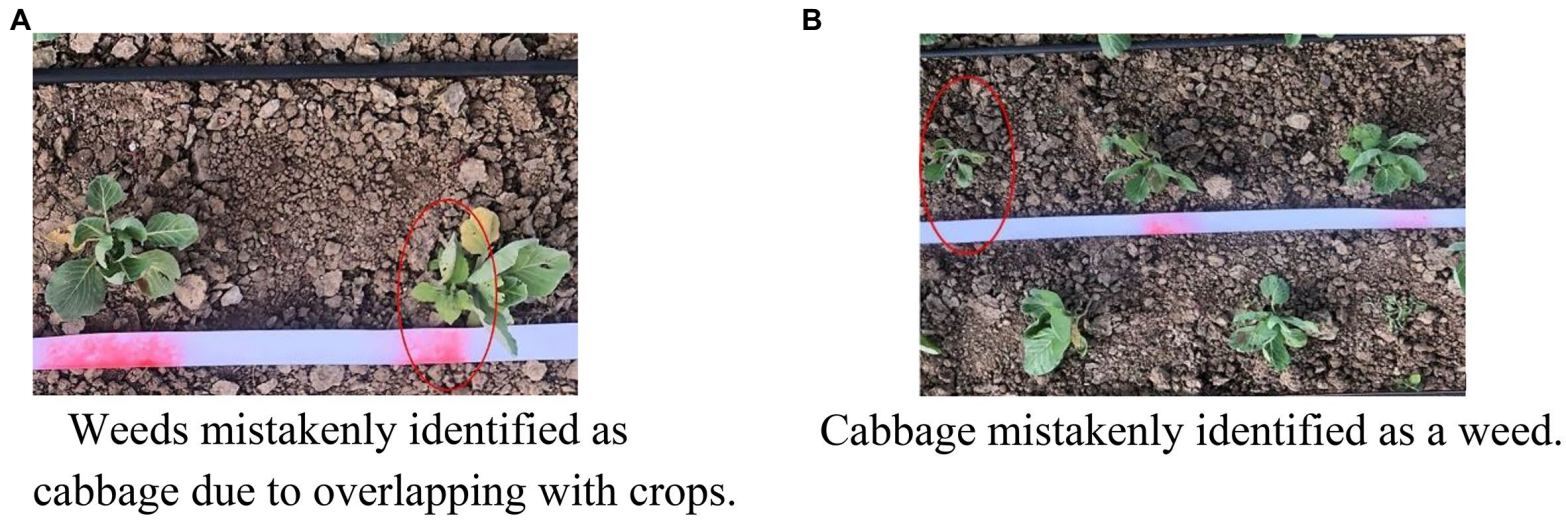
Actual target spraying effect in the field. **(A)** Weeds mistakenly identified as cabbage due to overlapping with crops. **(B)** Cabbage mistakenly identified as a weed.

Although most cabbage and weeds can be correctly identified during operation, there are still some cases where weeds are mistakenly identified as cabbage due to their own growth characteristics or because they partially overlap with crops (Figure 14A), which results in the destruction of the original features and incorrect identification. Furthermore, some cabbage samples are mistakenly identified and are not sprayed due to partial uneven or local reflections, which result in the destruction of the original features and incorrect identification, as shown in Figure 14B.

#### Relationship Between the Operation Speed and Effective Spraying

The corresponding statistical results of invalid spraying, missed spraying and effective spraying at the three speeds are shown in Table 6.

**Table 6:** Statistical results of targeted spraying at different speeds.

Operating speed/m·s^−1^	Number of targets	Number of invalid sprays	Invalid spraying rate/%	Number of missed sprays/个	Missed spraying rate/%	Number of effective sprayed targets/个	Effective spraying rate/%
0.52	304	4	1.3	14	4.6	286	94.1
0.69	313	6	1.9	12	3.8	295	94.3
0.93	324	13	4.0	18	5.6	293	90.4

**Figure 15:**
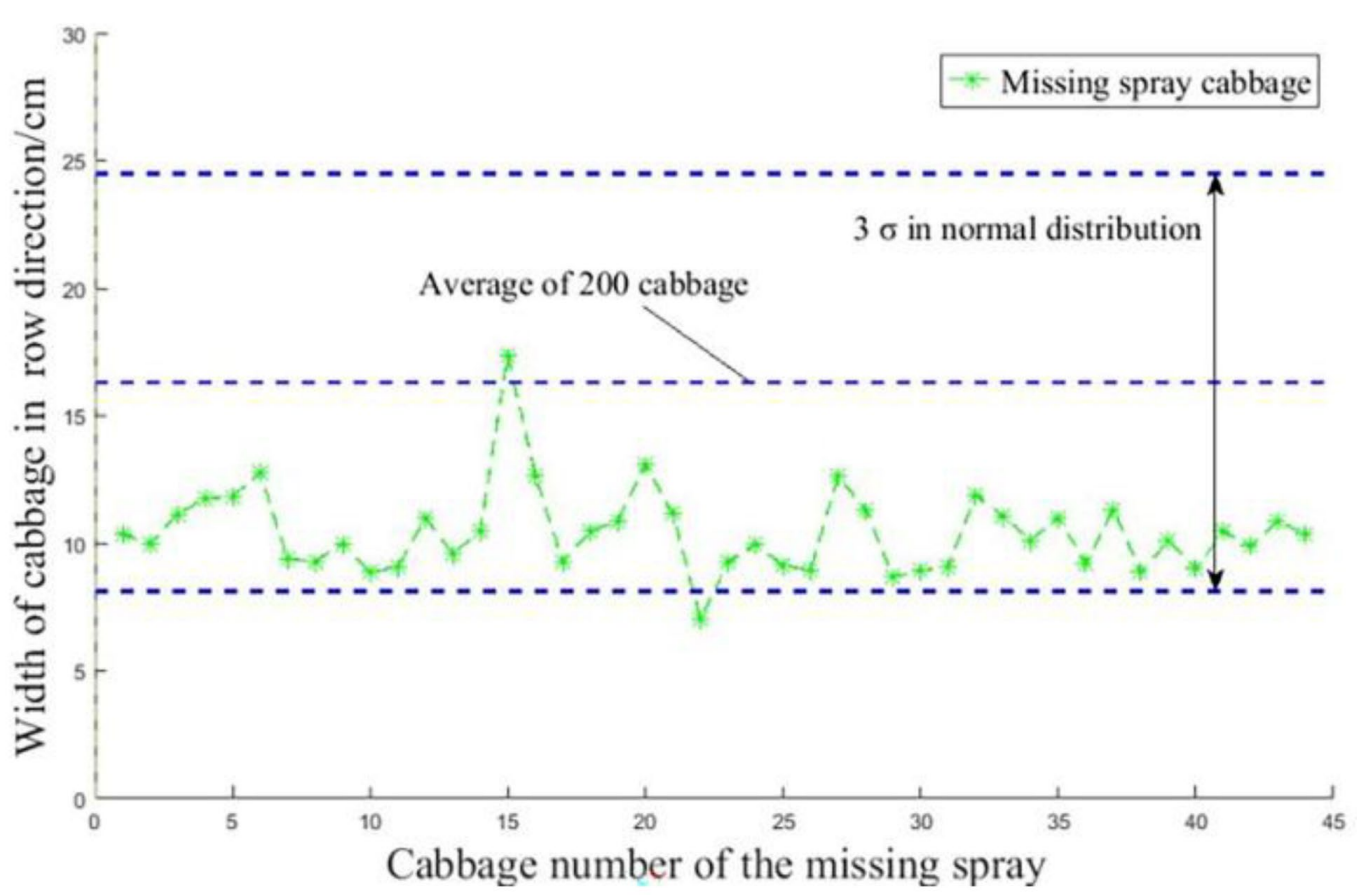
Size statistics of the missed targets.

At the three operating speeds, the highest invalid spraying rate, missed spraying rate, and effective spraying rate were 4.0, 5.6 and 94.3%, respectively, with an average invalid spraying rate, missed spraying rate and effective spraying rate of 2.4, 4.7, and 92.9%, respectively. When the operation speed increased from 0.52 to 0.93 m/s, the ineffective spraying rate continued to increase, indicating that the speed affects the effectiveness of spraying, which may be due to the speed affecting the accuracy of the opening or closing of the solenoid valve relative to the target position. Invalid spraying primarily occurred when the cabbage was partially recognized due to the influence of imaging factors in the real-time identification process or when the spraying position was obviously misaligned with the actual position of the target due to the influence of instantaneous skidding in the advancing process of the ground wheels. The missed spraying rate did not increase with increasing speed, indicating that there is no clear correlation between the missed spraying rate and the speed. As missed spraying means that there was no spraying action on the target, it can be believed that the solenoid valve did not open because the system did not identify the target or the identified target was too small. The reasons why a target was not recognized mainly include uneven, overlapping and occluded samples, as mentioned in Section “Image Acquisition.” When a target is identified as too small, it may be because the cabbage itself is small or that only parts of the cabbage are recognized due to local reflection or uneven light. To further analyse the relationship between cabbage size and the missed spray rate, the sizes of targets that were not sprayed were calculated, as shown in Figure 15.

According to the figure, it can be seen that the average size of the cabbage samples was 16.1 cm, and the average canopy size of 44 missed targets was 10.1 cm, among which the sizes of 43 missed targets were below 12.8 cm. The canopy size of 1 missed target was larger than the average value of 16.1 cm, indicating that the missed targets were mainly those with smaller canopy sizes, and the size of a target may affect the accuracy of identification; namely, the smaller the cabbage is, the lower the identification accuracy and the higher the missing spray rate.

#### Influence of the Operating Speed on the Target Offset

Absolute values were taken for the open distance, close distance and spray length of effectively sprayed targets at three speeds, and the average values were taken as the means. The statistical results are shown in Table 7.

**Table 7:** Results of the effective spraying of targets.

Speed/m·s^−1^	Effective spraying of targets					Mean of the spray length/mm	Proportional mean of the spray length/%	Deviation of the centre of mass/mm	Theoretical pesticide saving rate/%
	D_open/mm		D_close/mm						
	Mean	Standard deviation	Mean	Standard deviation					
0.52	40.1	12.2	38.2	11.4		220.5	65.1	14.1	45.4
0.69	42.0	20.7	35.4	20.3		219.4	64.7	16.4	44.9
0.93	64.1	28.5	11.1	31.0		210.5	60.6	28.6	47.4

According to the statistical results, at the three speeds, the mean spraying length was 216.8 mm, with a 1.6% error compared to the preset spraying length, the mean effective spraying length was 63.5%, and the theoretical pesticide saving rate was 45.9%. With increasing speed, the centre of the mass offset of the targets increased and reached a maximum value of 28.6 mm at 0.93 m/s. At speeds of 0.52 m/s and 0.69 m/s, the opening distance and closing distance were basically equal; that is, the better the target effect was, the smaller the centre of mass offset of the spray. At a speed of 0.93 m/s, the open distance increased and the close distance decreased, indicating that there was some skid in the advancing process of the wheels, and there was a certain offset between the encoder range and the theoretical target position; that is, the higher the speed was, the greater the skid rate of the ground wheels, and the larger the centre of mass offset of the spray. Similarly, the skid rate also affected the spraying length. When the distance calculated by the control system according to the encoder signal was larger than the actual operating distance, the spraying length was 210.5 mm at a speed of 0.93 m/s, which was significantly shorter than the spraying lengths at speeds of 0.52 m/s and 0.69 m/s. According to the speeds, it can be seen that with an increase in the vehicle speed, the standard deviation and the variance in the open distance and close distance increased gradually, indicating that an increase in speed results in a decrease in the matching accuracy between the crop spacing perceived by the nozzle controlled in real time and the actual spacing. Moreover, the uneven surface of the soil in the field also affected the position of the camera, which increased the difficulty in positioning crops and reduced the real-time matching accuracy between the crop coordinates and the spraying coordinates. As the advancing speed increased, under the condition that the response frequency of the relay and solenoid valve is certain, the spraying control error increased, resulting in a decrease in the proportion of the effective spraying length. If the preset open distance or close distance is increased, the proportion of the effective spraying length would also increase accordingly (Loghavi and Mackvandi, 2008). However, according to the theoretical definition of the pesticide saving rate, it can also be seen that the pesticide saving rate would decrease, that is, the longer the spraying length is, the lower the pesticide saving rate.

**Figure 16:**
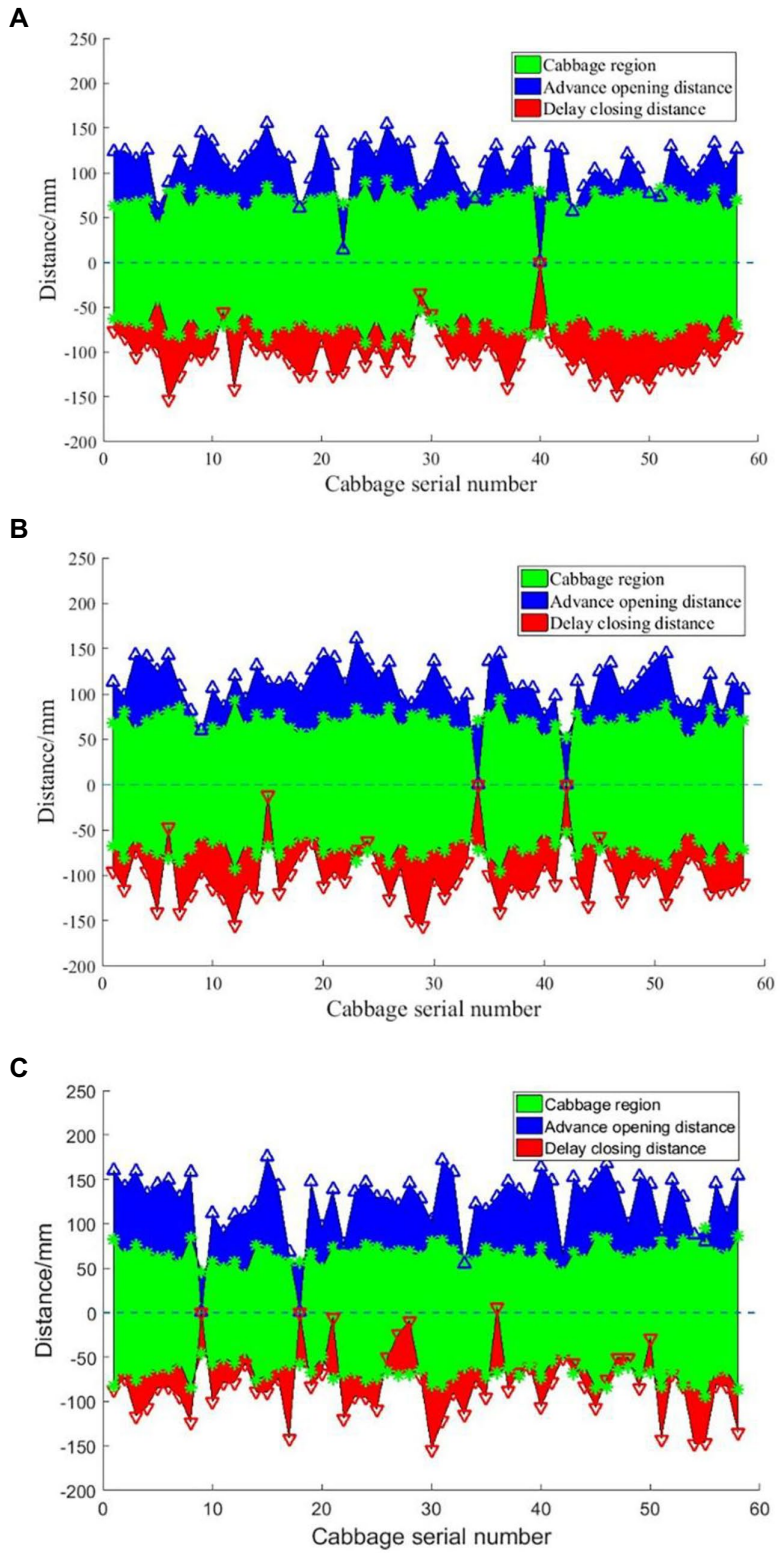
Statistical results of the continuous spraying of 58 cabbages at different speeds. **(A)** 0.52 m/s. **(B)** 0.69 m/s. **(C)** 0.93 m/s.

With the centre of a cabbage sample as the origin, the opening distance and closing distance of the nozzle relative to the centre of the cabbage sample were drawn, as shown in Figure 16A. At 0.52 m/s, the nozzle was delayed in opening and closing while spraying the 22nd cabbage, which may be due to the identification error caused by local reflection; thus, the first part of cabbage was not correctly identified, and only the second part of cabbage was sprayed. At 0.69 m/s, similar to the 15th cabbage, the latter part of the cabbage was not sprayed, as shown in Figure 16B. Furthermore, the size of the effectively identified sample limits the opening time of the solenoid valve. When the opening time is less than the response time of the solenoid valve, pesticide spraying may not be carried out by the system; that is, the missed spraying is not identified, such as for the 40th cabbage shown in Figure 16A, the 34th cabbage and 43rd cabbage and shown in Figure 16B, and the ninth cabbage and 18^th^ cabbage shown in Figure 16C. At a speed of 0.93 m/s, the fluctuation in the spraying distance increased significantly, and the number of cabbage samples partially sprayed or not sprayed significantly increased as well, as shown in Figure 16C.

#### Dosage Analysis of Targeted Pesticide Spraying

Table 8 shows the statistical results of the dosage of the targeted pesticide spraying and continuous spraying under the conditions of natural seeding deficiency and continuous growth. Under the condition of natural seeding deficiency, 941 cabbage samples in 8 ridges were tested, while under the condition of continuous growth, 320 cabbage samples over 124 m were tested. In addition, in the case of natural seeding deficiency in the field, the dosage and its standard deviation between each row of continuous spraying were 28.7 L and 0.09 L, while those of targeted spraying were 13.4 L and 0.2 L, indicating that the standard deviation of targeted spraying was significantly greater than that of continuous spraying, which mainly may be due to the inconsistent seedling deficiency in each row. Therefore, relatively large dosage differences among the targets occurred. Furthermore, compared with continuous spraying, under the condition of natural seeding deficiency, the targeted spraying dosage decreased by 15.3 L, with a savings rate of 53.3%, and under the condition of continuous growth, the targeted spraying dosage decreased by 2.4 L, with a savings rate of 33.8%. Additionally, there is a direct relationship between the pesticide saving rate and the canopy proportion (Berenstein and Edan, 2018). If the canopy proportion reaches a certain value, there would be no difference between target spraying and continuous spraying, and the effect of pesticide savings would not be achieved.

**Table 8:** Results of targeted pesticide spraying.

Condition	Void ratio/%	Number of actual targets	Continuous spraying		Target spraying		Saved pesticide volume/L	Pesticide saving rate/%
			Dosage/L	Standard deviation/L^2^	Dosage/L	Standard deviation/L^2^		
Natural seeding deficiency	76.6	941	28.7	0.09	13.4	0.2	15.3	53.3
Continuous growth	65.0	320	7.1	0.10	4.7	0.18	2.4	33.8

The targeted spray control system controls the opening and closing of multiple nozzles in real time according to the speed and the size of the targets and stabilizes the pressure by controlling the pressure fluctuation caused by the instantaneous opening and closing of nozzles to maintain the pressure fluctuations within a certain range. However, spraying flow is regulated by the pressure, the actual supply pressure of each solenoid valve of targeted pesticide spraying is different, and the flow change depends more on the distribution of the nozzles (Han et al., 2001). In the future, on the basis of the targeted pesticide spraying model proposed in this study, further research on the pressure in the pesticide supply system as well as the distribution of the flow will be carried out to improve the dosage of targeted pesticide spraying and achieve better effects while saving pesticides.

## Conclusion

A targeted pesticide spraying control system based on an active light source and a targeted spraying delay model are designed to prevent the influence of real-time changes in natural lighting on target identification. A communication protocol for the targeted spraying control system is developed, in which target identification and positioning are carried out by video stream, and the positioning distance is calculated by encoder ranging to realize the precise control of targeted spraying. Real-time cabbage target identification is realized based on an SVM, and a concept of taking the skeleton point-to-line ratio and ring structure characteristics as the classification and identification characteristics of the SVM is proposed to classify and test the different characteristic combinations of the SVM. From this, the characteristic vector comprised of the point-to-line ratio, maximum inscribed circle radius, and fitted curve coefficient has the highest identification rate of up to 95.7%, and its test time is 33 ms, which meets the needs of actual production. Field tests are carried out to verify the identification accuracy and control accuracy of the target spraying machine, in which the identification accuracy of cabbage samples is 95.0%, the range of the identification accuracy of various weeds is 92.2–96.2%, and the average identification accuracy is 93.5%. In addition, under three operation speeds, the average invalid spraying rate, average missed spraying rate, and average effective spraying rate are 2.4, 4.7, and 92.9%, respectively. With increasing speed, the offset of the centre of the mass of the target increases and reaches a maximum value of 28.6 mm when the speed is 0.93 m/s. Under natural seeding deficiency conditions, the void rate and pesticide saving rate were 65 and 33.8%, respectively, while they were 76.6 and 53.3%, respectively, under continuous growth conditions.

## Data Availability Statement

The raw data supporting the conclusions of this article will be made available by the authors, without undue reservation.

## Author Contributions

XZ performed most of the experiments with the assistance of HF and SY. CL designed the software. XZ, CZ, and XW designed the study, analysed the data, and wrote the manuscript. All authors contributed to the article and approved the submitted version.

## Funding

This research was funded by the Special Project of Strategic Leading Science and Technology of Chinese Academy of Sciences (XDA28090108), National Modern Agricultural Industrial Technology System Construction Project (KFZN2021W001), Special Project for Innovation Capacity Building of Beijing Academy of Agricultural and Forestry Sciences (KJCX20210402), and the National Key Research and Development Program of China (2019YFE0125200).

## Conflict of Interest

The authors declare that the research was conducted in the absence of any commercial or financial relationships that could be construed as a potential conflict of interest.

## Publisher’s Note

All claims expressed in this article are solely those of the authors and do not necessarily represent those of their affiliated organizations, or those of the publisher, the editors and the reviewers. Any product that may be evaluated in this article, or claim that may be made by its manufacturer, is not guaranteed or endorsed by the publisher.

## Supplementary Material

The Supplementary Material for this article can be found online at: https://www.frontiersin.org/articles/10.3389/fpls. 2022.924973/full#supplementary-material
